# Adaptation strategy of karst forests: Evidence from the community‐weighted mean of plant functional traits

**DOI:** 10.1002/ece3.8680

**Published:** 2022-03-01

**Authors:** Libin Liu, Jing Hu, Xinyao Chen, Xin Xu, Yong Yang, Jian Ni

**Affiliations:** ^1^ College of Chemistry and Life Sciences Zhejiang Normal University Jinhua China; ^2^ State Key Laboratory of Environmental Geochemistry Institute of Geochemistry Chinese Academy of Sciences Guiyang China

**Keywords:** community level, ecological stoichiometry, forest restoration, interspecific variation, karst terrain, morphological trait, survival strategy

## Abstract

Conservative survival strategy of plants growing in harsh karst habitats is observed from the view of plant functional traits, such as morphological traits and ecological stoichiometry. However, whether the plant communities in karst forests with high species turnover adopt a conservative strategy remains undetermined. This study comprehensively investigated the characteristics of functional traits of dominant plant species in four forests (i.e. *Platycarya strobilacea*, *Quercus fabri*, *Quercus variabilis*, and *Pinus massoniana* forests) in a trough‐valley karst watershed in Northern Guizhou Province, Southwestern China to explore the adaptation strategy of karst forests at the community level. At the organ and the species levels, traits differed among species, and the leaf and the bark morphological traits and root C:N:P ecological stoichiometry presented large interspecific variations. At the community level, the *P*. *massoniana* forest presented the lowest specific root length and dry matter content and tissue density of roots, branch, twig, and bark; the *Q*. *fabri* and the *Q*. *variabilis* forests displayed low specific leaf area and high dry matter content and tissue density of roots, branch, and twig; and the *Platycarya strobilacea* forest exhibited high specific leaf area. The *P*. *massoniana* forest was subjected to N and P colimitation, and the three other broad‐leaved forests were limited by P supply. The community‐weighted means rather than the arithmetic means of traits were preferential to represent the trait characteristics at the community level. From the view of plant functional traits at the community level, karst forests develop multiple functional traits like low specific leaf area, high dry matter content and tissue density of leaf, roots, branch, and twig, and decrease N and P investments in leaf for a conservative survival strategy to adapt to harsh habitats.

## Graphical abstract

This manuscript is about the comprehensive investigation of 18 morphological traits of leaf, root, branch, twig, and bark and C:N:P ecological stoichiometry of leaf, root, and branch of dominant species in four karst forests (i.e., *Platycarya strobilacea*, *Quercus fabri*, *Quercus variabilis*, and *Pinus massoniana* forests) in a trough‐valley karst terrain in Northern Guizhou Province, Southwestern China. Results show that the community‐weighted means rather than the arithmetic means of traits were preferential to represent the trait characteristics at the community level. From the view of plant functional traits at the community level, karst forests develop multiple functional traits like low specific leaf area, high dry matter content and tissue density of leaf, roots, branch, and twig, and decrease N and P investments in leaf for a conservative survival strategy to adapt to harsh habitats.

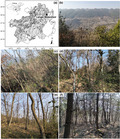

## 1 INTRODUCTION

Plant functional traits (PFTs) are the inherently physiological and externally morphological characteristics highly related to the ecesis, survival, growth, and death processes of plants (Violle et al., 2007). The trait assembly of the different organs of plants can embody their resource acquisition and allocation strategies and reflect the functional characteristics of ecosystems (Díaz & Cabido, 2001; Garnier et al., 2004). Thus, PFTs play important roles in connecting plant individuals with environments and ecosystem structures, processes, and functions (Koerselman & Meuleman, 1996; McGill et al., 2006; Westoby & Wright, 2006). The study of PFTs provides another pathway to understand the population survival strategy, biodiversity maintenance, biological invasion, and vegetation modeling (Díaz & Cabido, 1997; Huang et al., 2016; Kraft et al., 2008; Sutherland, 2004; Wang et al., 2017).

Most PFT studies worldwide focus on the organ and the species levels, whereas PFT studies conducted at the community and the ecosystem levels are often underpowered (He et al., 2018; Zhang et al., 2018). Furthermore, the arithmetic means of several dominant species are used to represent the community trait values. Such data analysis may bring about remarkable uncertainties, and results may not reflect the traits of a plant community. Natural plant communities are composed of species adapted to certain environments, and different species play different roles in community assembly and function exertion (Grime, 1998; Huston, 1997). Arithmetic mean trait values evidently fail to consider the complexity of species composition, community structures, and functions in complex natural plant communities (Díaz et al., 2016; Muscarella & Uriarte, 2016; Wright et al., 2004). Besides, no criterion is available in the selection of dominant species and individuals, for example, the number of species that should be chosen. Thus, PFT investigations that consider species composition, community structures, and functions at the plant community level must be conducted.

Karst, an extremely unique geomorphology that has resulted from the solvation of carbonatite (limestone and dolomite) is sporadically ubiquitous in the global land area but widespread around the southern United States, Mediterranean coasts of Europe, and Southwestern China (Jiang et al., 2014). In Southwestern China, vegetation degradation happens everywhere due to the fragility of karst ecosystems and intensive human disturbances. The forest restoration of degraded vegetation has become an environmental topic in karst regions. PFTs and the trait‐based community ecology theory (a theory using trait‐based approaches to determine community composition, structures, and functions) can reveal the adaptation strategies of vegetation in different restoration stages and environmental habitats and evaluate the restoration effects of different modes (Hedberg et al., 2013; Lavorel & Garnier, 2002; Pywell et al., 2003; Roberts et al., 2010; Sandel et al., 2011). Existing research on PFTs in Southwestern China indicates that plants grow in a plateau‐surface, peak‐clum depression, and peak‐forest plain karst morphological terrains with harsh habitats (e.g., high temperature, water shortage, and shallow soils) exhibit low leaf area (LA), specific leaf area (SLA), and fine root‐specific length (SRL), high leaf dry matter content (LDMC), and leaf tissue density (LTD). Plant growth is limited by N and P supply, and the interspecific variations of PFTs are generally large (Jiang et al., 2016; Liu et al., 2014, 2015, 2019; Pang et al., 2019; Pi et al., 2017; Yang et al., 2020; Zhong et al., 2018). As a result, the conservative survival strategy with low growth rate and high resource utilization of karst plants is commonly observed (Tang et al., 2016).

However, most previous PFT studies in karst areas focus on leaf traits, and traits of other organs (root, branch, trunk, and bark) are rarely reported (Liu et al., 2019; Yang et al., 2020; Zhong et al., 2018). Furthermore, nearly all previous PFT studies stay at the organ and the species levels. The arithmetic mean trait values of the chosen species are treated as the community trait values (Xi et al., 2011). Such community trait values may be accompanied by significant uncertainties caused by large interspecific variations of traits, and biomass and individual number differences of the chosen species in complex natural plant communities. For example, among the chosen five dominant tree species in a karst secondary forest in Central Guizhou Province, *Carpinus pubescens* presents considerably lower leaf thickness (LT) and LA, considerably higher SLA, lowest biomass stock, and smallest individual number; *Lithocarpus confinis* displays considerably lower leaf N and P contents, highest biomass stock and largest individual number. The arithmetic mean and community‐weighted mean (CWM, calculated on the basis of the relative biomass or individual number) trait values of the forest would differ considerably (Liu et al., 2019; Zhong et al., 2018). Zhang et al. (2018) have also found that the CWM (calculated on the basis of the relative biomass) and the arithmetic mean values of C:N:P ecological stoichiometry in China's forests differ remarkably, and the former is better to represent the ecological stoichiometry at the community level. Therefore, the CWM of traits of the leaf together with other organs may reflect the community trait characteristics and reveal the adaptation strategy of karst plants at the community level.

In the present study, three natural secondary forests and an artificial forest with different restoration years in a trough‐valley karst watershed in Southwestern China are investigated as examples. Eighteen morphological traits of leaf, root, branch, twig, and bark and the C:N:P ecological stoichiometry of leaf, root, and branch of dominant species are comprehensively determined, and the CWM values of all traits are further calculated on the basis of the relative biomass. Does this study aim to answer what adaptation strategy do forests growing in harsh karst habitats adopt from the view of PFTs at the community level? Specifically, this study tests the following predictions: (1) karst plant species present large interspecific variations in PFTs; (2) the CWM and arithmetic mean trait values display great differences in karst forests; and (3) karst forests adopt conservative survival strategy with low growth rate and high resource utilization. Such a study will broaden the understanding of the vegetation–environment interactions and guide the ecological restoration in karst regions in Southwestern China.

## 2 MATERIALS AND METHODS

### 2.1 Study area

**FIGURE 1:**
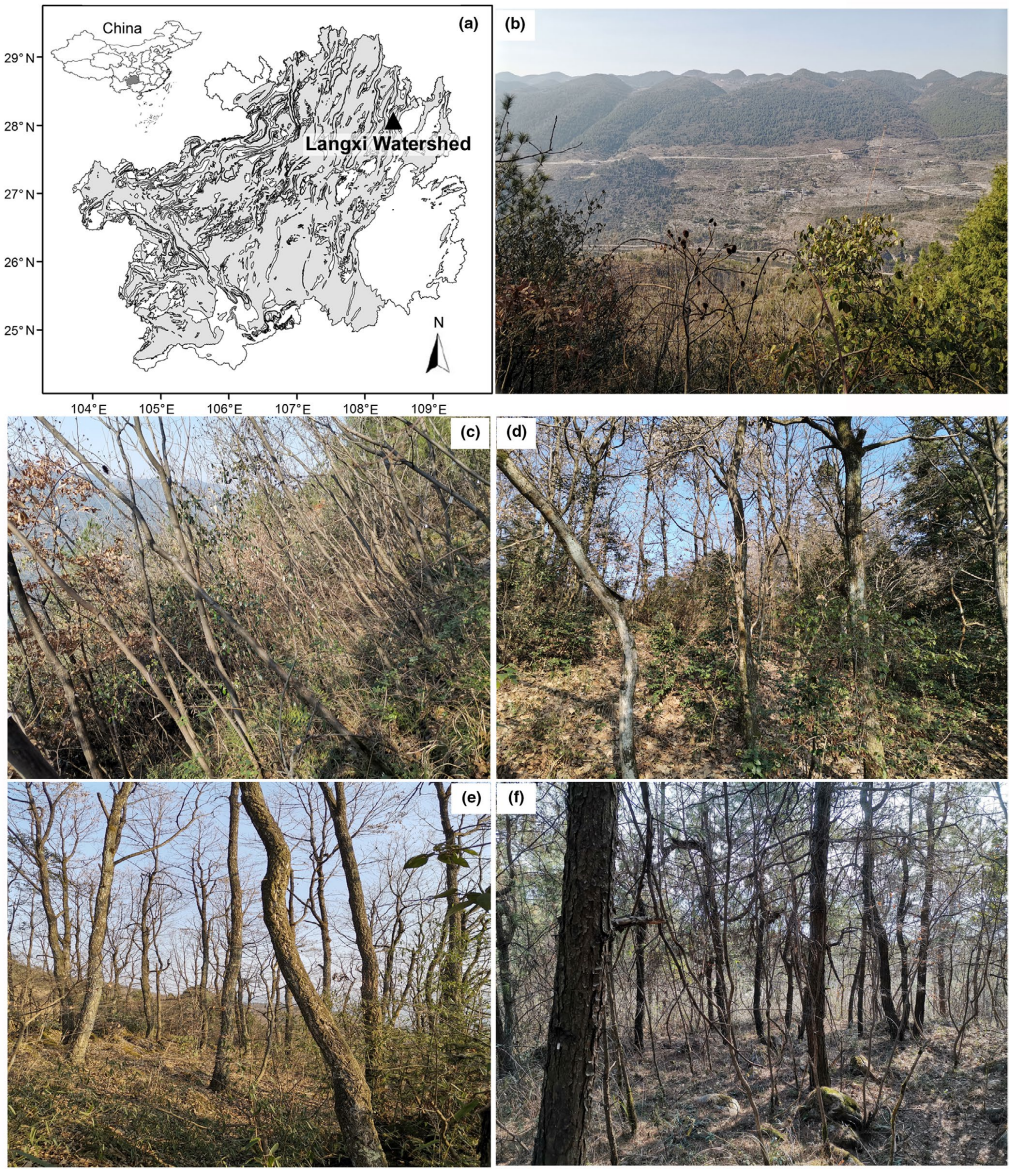
Location (a) and physiognomy (photographed in winter) of the Langxi Watershed (b) and the four karst forests (c): *Platycarya strobilacea* forest, (d): *Quercus fabri* forest, (e): *Quercus variabilis* forest, (f): *Pinus massoniana* forest) in the distribution map of karst terrain (the gray) in Guizhou Province, Southwestern China

The Langxi Watershed in Yinjiang County, a typical and representative basin in the trough‐valley karst morphological terrain, is located in Northern Guizhou Province, Southwestern China (Figure 1). This terrain lies in mid‐subtropical China and has a monsoon climatic regime. According to records from the Yinjiang weather station (108°24′ E, 28°01′ N, 457 m) in 1961–2009, the mean annual air temperature is 16.8°C, with the lowest monthly mean in January (5.6°C) and the highest monthly mean in July (27.0°C). The mean annual precipitation is 1114.7 mm, of which 68.7% occurs between April and August. The mean annual sunshine duration is 1222.8 h, with a low sunshine percentage of 25.5%. The parent rock is limestone, and the dominant soil is yellow limestone soil (Yang et al., 2020). The native vegetation in the Langxi Watershed has been destroyed. Degraded shrublands and grasslands, man‐made orchard lands, and rice fields are distributed at the foot and the middle of mountains. Natural secondary forests, including *Platycarya strobilacea* forest (regenerated from an abandoned land in 1992), *Quercus fabri* forest (regenerated from a clear cutting in 1978), *Quercus variabilis* forest (regenerated from a clear cutting in 1958), and some other broad‐leaved forests with small areas, and artificial coniferous forest (*Pinus massoniana* forest, planted in 1968) are only distributed in mountaintops with less human disturbances (Figure 1) (Yang et al., 2020).

### 2.2 Vegetation survey and biomass estimation

After complete vegetation investigations in the watershed, four plots (each with an area of 50 m × 50 m) of the four dominant forest types were established (Table 1). Each woody plant with a diameter at breast height (*D*) ≥1 cm was recorded with species identity (botanical nomenclature was based on Chen, 1982–2004), *D* (measured using a diameter tape), height (measured using a telescopic rod and a steel tape), and canopy width (canopy projection width, measured using a steel tape). The total biomass of each individual was estimated using biomass allometric models (Table S1). The biomass of tree species with ≥15 individuals in each plot was estimated using their own biomass allometric models, and the biomass of other tree and shrub species was estimated using universal allometric models (Liu et al., 2020).

**TABLE 1:** Basic information of four karst forest plots in Northern Guizhou Province, Southwestern China

Items	*Platycarya strobilacea* forest	*Quercus fabri* forest	*Quercus variabilis* forest	*Pinus massoniana* forest
Location	108°30ʹ15″ E, 28°02ʹ 12″ N	108°25ʹ29″ E, 27°56ʹ54″ N	108°25ʹ32″ E, 27°57ʹ02″ N	108°25ʹ29″ E, 27°56ʹ34″ N
Elevation (m)	916	1193	1186	1243
Rock coverage (%)	17.59	11.24	24.70	25.92
Soil thickness (cm)	57.75	50.24	66.63	60.72
Stand age	25	40	60	50
Species richness	39	29	49	38
Stand density (individuals/hm^2^)	10908	7452	4320	4884
Average diameter at breast height (cm)	2.86 ± 2.41	4.35 ± 6.08	5.03 ± 8.16	5.48 ± 5.92
Height of tree layer (m)	8–11	7–11	15–21	8–14
Common species	*P*. *strobilacea*, *P*. *massoniana*, *Albizia kalkora*, *Platycladus orientalis*	*Q*. *fabri*, *Quercus acutissima*, *Camellia japonica*, *P*. *massoniana*	*Q. variabilis*	*P*. *Massoniana*, *Lindera glauca*

In each forest, the species chosen for PFT measurements accounted for not less than 90% of the total forest biomass. According to biomass distribution patterns among species in the four karst forests, nine species, that is, *P*. *strobilacea* (accounting for 60.92% of the forest biomass), *P*. *massoniana* (16.65%), *Albizia kalkora* (7.52%), and *Platycladus*. *orientalis* (7.13%) in *P*. *strobilacea* forest; *Q*. *fabri* (57.55%), *Quercus acutissima* (24.22%), *Camellia japonica* (5.23%), and *P*. *massoniana* (3.56%) in *Q*. *fabri* forest; *Q*. *variabilis* (95.09%) in *Q*. *variabilis* forest and *P*. *massoniana* (86.20%) and *Lindera glauca* (3.80%) in *P*. *massoniana* forest, were chosen.

### 2.3 Measurement of morphological traits

Twenty healthy dominant individuals per species in each forest were selected. Four branches were collected from four different positions of the sunlit side of the tree canopy in each sampled individual. Five healthy mature leaves (10 healthy mature needles) without visible damage of each branch were collected. An approximately 20 cm length terminal twig and an approximately 5 cm length branch (diameter ≥1 cm) were sampled from one of the four branches. A taproot of each individual was dug out, and roots were separated into coarse (root diameter ≥10 mm), medium (root diameter = 2–10 mm), and fine (root diameter ≤2 mm) roots. A bark sample at the *D* position of each individual was collected.

Fresh masses of leaf, root, branch, twig, and bark samples were weighed using an electronic balance (accurate to 0.001 g). Bark thickness (BaT, mm) and LT (mm) values were measured using an electronic Vernier caliper (accurate to 0.01 mm). The LA, fine root length, and volume were scanned using the WinFOLIA multipurpose leaf area meter (Regent Instruments, Canada) (Yang et al., 2020; Zhong et al., 2018). The volumes of coarse and medium roots, branch, twig, and bark samples were determined using the drainage method, and those of leaf samples were obtained as the product of LA and LT (Cornelissen et al., 2003). All samples were dried at 85°C for 72 h in an oven to determine their dry masses. The values of morphological traits were calculated as shown in Table S2.

### 2.4 Determination of elemental contents

After morphological trait measurements, 5 leaves, 5 roots (mixed with coarse, medium, and fine roots), and 5 branch samples of each species were selected. All plant samples were powdered and sieved through a 0.2 mm sieve. The contents of total C (TC) and total N (TN) of the leaf (LC and LN), root (RC and RN), and branch (BrC and BrN) were determined using the Vario MACRO Cube (Thermo Scientific, Germany), and those of total P (TP) of the leaf (LP), root (RP), and branch (BrP) were determined using the iCAP 6300 ICP‐OES Spectrometer Analyzer (Thermo Scientific, USA).

### 2.5 Data analysis

In accordance with empirical studies (He et al., 2019; Zhang et al., 2018), the relative biomass (i.e., the biomass of one species as a percentage of the total forest biomass in each plot) was used to extrapolate PFTs from the species level to the community level. The CWM of a single trait was treated as the average trait value in the community, and was calculated using the following equation:

$$\text{CWM}x=\left(\sum_{i=1}^{s}Bi\times ti\right)/Bs,$$

where CWM*_x_* is the CWM for trait *x*; *s* is the number of species, which accounts for not less than 90% of the total biomass in the forest community; *B_i_* is the relative biomass of the *i*th species in the forest community; *t_i_* is the trait value for the *i*th species, and *B_s_* is the biomass percentage of the chosen species in the forest community.

The coefficients of interspecific variation (standard deviation divided by mean) were used to characterize the varying degrees of PFTs among plant species. LN, LP, and leaf N/P ratio (LN/P) were used as indicators to compare the resource utilization between karst forests and plants in China and in the world. The one‐sample *t*‐test was conducted to determine differences between average LN and LP of plants in China and in the world and corresponding elemental contents of the four karst forests. The principal component analysis (PCA) was done to evaluate the effects of plant species and forest type on PFTs, and show the distributions of the PFTs among plant species and forest type. Trait data were log‐transformed prior to PCA analysis. All statistical analyses were performed using the SPSS version 20 and the CANOCO 5 (ter Braak & Smilauer, 2012; Xue, 2017).

## 3 RESULTS

### 3.1 Morphological traits of plant species

**FIGURE 2:**
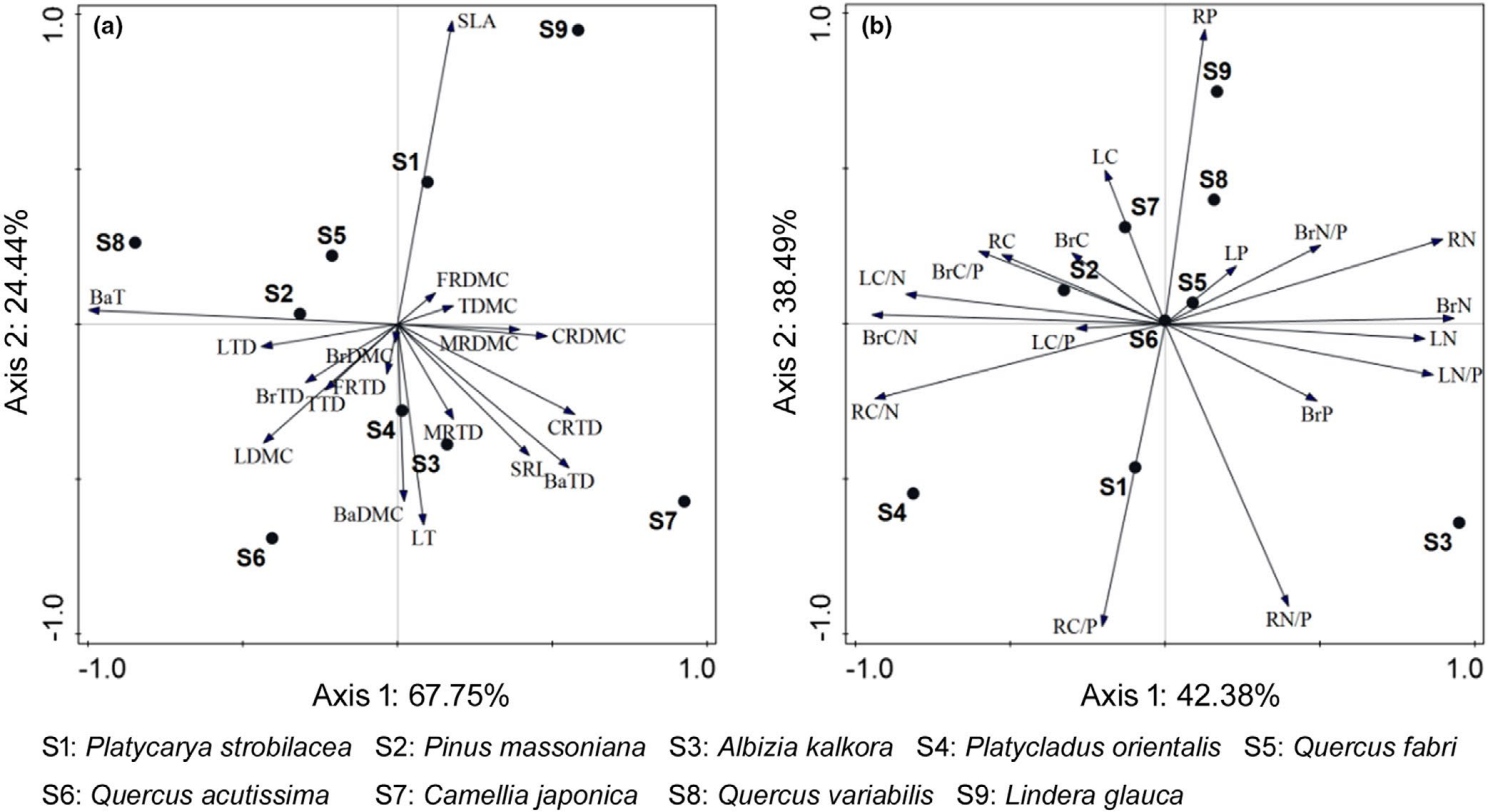
PCA showing the distribution of the morphological traits (a) and ecological stoichiometry (b) among dominant species in karst forests in Northern Guizhou Province, Southwestern China. Axis1 accounted for 67.75% (a) or 42.38% (b) of the variables, and Axis2 accounted for 24.44% (a) or 38.49% (b) of the variables. LT, leaf thickness; LTD, leaf tissue density; LDMC, leaf dry‐matter content; SLA, specific leaf area; CRTD, coarse root tissue density; CRDMC, coarse root dry‐matter content; MRTD, medium root tissue density; MRDMC, medium root dry‐matter content; FRTD, fine root tissue density; FRDMC, fine root dry‐matter content; SRL, fine root specific length; BrTD, branch tissue density; BrDMC, branch dry‐matter content; TTD, twig tissue density; TDMC, twig dry‐matter content; BaT, bark thickness; BaTD, bark tissue density; BaDMC, bark dry‐matter content; LC, leaf total carbon content; LN, leaf total nitrogen content; LP, leaf total phosphorus content; LC/N, leaf carbon–nitrogen ratio; LC/P, leaf carbon–phosphorus ratio; LN/P, leaf nitrogen–phosphorus ratio; RC, root total carbon content; RN, root total nitrogen content; RP, root total phosphorus content; RC/N, root carbon–nitrogen ratio; RC/P, root carbon–phosphorus ratio; RN/P, root nitrogen–phosphorus ratio; BrC, branch total carbon content; BrN, branch total nitrogen content; BrP, branch total phosphorus content; BrC/N, branch carbon–nitrogen ratio; BrC/P, branch carbon–phosphorus ratio; BrN/P, branch nitrogen–phosphorus ratio

Morphological traits varied among plant species (Figure 2a, Table 2). *P*. *massoniana* (the artificial species) dominated low values of traits. Twelve (i.e., LDMC, CRTD: coarse root tissue density, CRDMC: coarse root dry matter content, MRTD: medium root tissue density, MRDMC: medium root dry matter content, FRTD: fine root tissue density, FRDMC: fine root dry matter content, SRL, BrTD: branch tissue density, BrDMC: branch dry matter content, TTD: twig tissue density and BaTD: bark tissue density) of the 18 traits of *P*. *massoniana* were the lowest. *P*. *strobilacea* and *A*. *kalkora* showed high DMC and TD of the coarse, medium, and fine roots. *Q*. *acutissima* exhibited the highest LTD, LDMC, BrTD, BrDMC, and TTD and the lowest SLA. *Q*. *variabilis* presented the highest BaT. *L*. *glauca* displayed the highest SLA and the lowest LT. *C*. *japonica* had the highest LT, SRL, TDMC (twig dry matter content), BaTD, and BaDMC (bark dry matter content), and the lowest LTD and BaT. *P*. *orientalis* and *Q*. *fabri* presented intermediate trait values (Table 2).

**TABLE 2:** Morphological traits (mean ± SD) and coefficient of variation in dominant plant species in four karst forests in Northern Guizhou Province, Southwestern China

Plant functional Traits	*Platycarya strobilacea*	*Pinus massoniana*	*Albizia kalkora*	*Platycladus orientalis*	*Quercus fabri*	*Quercus. acutissima*	*Camellia japonica*	*Quercus variabilis*	*Lindera glauca*	Mean
LT (mm)	0.14 ± 0.03	0.41 ± 0.15	0.16 ± 0.02	0.29 ± 0.05	0.22 ± 0.03	0.27 ± 0.05	0.47 ± 0.02	0.21 ± 0.02	0.08 ± 0.03	0.25 ± 0.13 (50.23%)
LTD (g/cm^3^)	0.53 ± 0.06	0.60 ± 0.10	0.43 ± 0.05	0.45 ± 0.06	0.40 ± 0.05	0.85 ± 0.24	0.33 ± 0.03	0.47 ± 0.07	0.54 ± 0.18	0.51 ± 0.15 (29.64%)
LDMC (g/g)	0.49 ± 0.03	0.39 ± 0.02	0.44 ± 0.02	0.42 ± 0.04	0.48 ± 0.04	0.81 ± 0.07	0.40 ± 0.02	0.48 ± 0.07	0.40 ± 0.08	0.48 ± 0.13 (26.94%)
SLA (cm^2^/g)	143.13 ± 17.61	86.33 ± 12.54	60.23 ± 6.76	69.55 ± 9.21	117.23 ± 13.93	46.64 ± 9.50	65.93 ± 7.34	104.28 ± 18.52	252.33 ± 90.32	105.07 ± 63.10 (60.05%)
CRTD (g/cm^3^)	0.58 ± 0.07	0.38 ± 0.06	0.66 ± 0.06	0.52 ± 0.09	0.51 ± 0.14	0.58 ± 0.08	0.63 ± 0.06	0.49 ± 0.07	0.56 ± 0.06	0.54 ± 0.08 (15.38%)
CRDMC (g/g)	0.56 ± 0.10	0.41 ± 0.06	0.48 ± 0.12	0.50 ± 0.05	0.50 ± 0.13	0.55 ± 0.06	0.56 ± 0.04	0.48 ± 0.05	0.53 ± 0.04	0.51 ± 0.05 (9.52%)
MRTD (g/cm^3^)	0.56 ± 0.07	0.32 ± 0.10	0.61 ± 0.10	0.49 ± 0.12	0.53 ± 0.12	0.58 ± 0.10	0.54 ± 0.08	0.50 ± 0.09	0.47 ± 0.04	0.51 ± 0.09 (16.74%)
MRDMC (g/g)	0.57 ± 0.06	0.38 ± 0.11	0.58 ± 0.06	0.52 ± 0.09	0.53 ± 0.07	0.56 ± 0.08	0.55 ± 0.07	0.51 ± 0.07	0.57 ± 0.03	0.53 ± 0.06 (11.80%)
FRTD (g/cm^3^)	0.71 ± 0.15	0.39 ± 0.07	0.73 ± 0.11	0.60 ± 0.59	0.47 ± 0.12	0.66 ± 0.120	0.54 ± 0.14	0.64 ± 0.11	0.55 ± 0.09	0.59 ± 0.11 (18.88%)
FRDMC (g/g)	0.64 ± 0.08	0.46 ± 0.11	0.63 ± 0.22	0.52 ± 0.14	0.53 ± 0.09	0.63 ± 0.08	0.58 ± 0.13	0.63 ± 0.07	0.63 ± 0.23	0.58 ± 0.06 (11.11%)
SRL (cm/g)	18.09 ± 4.52	16.42 ± 3.92	17.80 ± 9.72	21.94 ± 11.30	27.30 ± 13.92	25.90 ± 8.50	32.32 ± 17.52	17.91 ± 13.36	20.60 ± 10.29	22.03 ± 5.39 (24.47%)
BrTD (g/cm^3^)	0.57 ± 0.06	0.46 ± 0.06	0.51 ± 0.07	0.60 ± 0.05	0.66 ± 0.04	0.72 ± 0.04	0.64 ± 0.033	0.72 ± 0.02	0.57 ± 0.04	0.61 ± 0.09 (15.15%)
BrDMC (g/g)	0.56 ± 0.04	0.45 ± 0.05	0.57 ± 0.04	0.55 ± 0.02	0.64 ± 0.03	0.66 ± 0.03	0.61 ± 0.02	0.63 ± 0.08	0.61 ± 0.02	0.59 ± 0.06 (10.66%)
TTD (g/cm^3^)	0.54 ± 0.08	0.43 ± 0.06	0.44 ± 0.14	0.66 ± 0.08	0.66 ± 0.05	0.68 ± 0.05	0.583 ± 0.064	0.62 ± 0.11	0.52 ± 0.04	0.57 ± 0.09 (16.25%)
TDMC (g/g)	0.53 ± 0.06	0.49 ± 0.08	0.47 ± 0.07	0.56 ± 0.02	0.67 ± 0.16	0.67 ± 0.05	0.69 ± 0.03	0.62 ± 0.08	0.68 ± 0.09	0.60 ± 0.09 (14.47%)
BaT (mm)	4.04 ± 0.90	8.19 ± 8.85	3.15 ± 1.159	4.44 ± 1.38	7.62 ± 2.46	9.61 ± 3.11	0.29 ± 0.10	22.15 ± 5.46	1.42 ± 0.40	6.77 ± 6.56 (96.89%)
BaTD (g/cm^3^)	0.49 ± 0.04	0.32 ± 0.08	0.45 ± 0.04	0.42 ± 0.04	0.51 ± 0.04	0.72 ± 0.08	0.10 ± 0.13	0.42 ± 0.09	0.51 ± 0.04	0.54 ± 0.20 (37.91%)
BaDMC (g/g)	0.50 ± 0.06	0.59 ± 0.06	0.45 ± 0.03	0.58 ± 0.04	0.59 ± 0.02	0.66 ± 0.03	0.78 ± 0.05	0.62 ± 0.07	0.46 ± 0.02	0.58 ± 0.10 (17.69%)

Abbreviatins: LT, leaf thickness; LTD, leaf tissue density; LDMC, leaf dry‐matter content; SLA, specific leaf area; CRTD, coarse root tissue density; CRDMC, coarse root dry‐matter content; MRTD, medium root tissue density; MRDMC, medium root dry‐matter content; FRTD, fine root tissue density; FRDMC, fine root dry‐matter content; SRL, fine root‐specific length; BrTD, branch tissue density; BrDMC, branch dry‐matter content; TTD, twig tissue density; TDMC, twig dry‐matter content; BaT, bark thickness; BaTD, bark tissue density; BaDMC, bark dry‐matter content.

In general, the morphological traits of leaf and bark presented large interspecific variations as shown by large coefficients of interspecific variation; and those of roots, branch, and twig showed small interspecific variations as indicated by small coefficients of interspecific variation (Table 2). The maximum coefficient of interspecific variation was BaT (96.89%. Table 2). SLA (60.05%) and LT (50.23%) also presented relatively large coefficients of interspecific variation (Table 2). CRDMC presented the minimum coefficient of interspecific variation (9.52%, Table 2).

### 3.2 Ecological stoichiometry of plant species

Ecological stoichiometry differed among plant species (Figure 2B, Table 3). *P*. *massoniana* exhibited the highest and *P*. *strobilacea* exhibited the lowest LC and RC. *A*. *kalkora* showed the highest LN, RN, BrN, LN/P, root N/P ration (RN/P), and BrP and the lowest leaf, root and branch C/N ratios (LC/N, RC/N, and BrC/N) and branch C/P ratio (BrC/P). *P*. *orientalis* presented the highest RC/N, BrC/N, BrC/P, root C/P ratio (RC/P), and the lowest LN/P, RN, RP, and BrN. *C*. *japonica* displayed the highest LC/N and leaf C/P ratio (LC/P) and the lowest LN, LP, and branch B/N ratio (BrN/P). *Q*. *variabilis* had the highest BrN/P and the lowest BrP. *L*. *glauca* showed the highest LP, RP, and BrC and the lowest LC/P, RC/P, and RN/P. *Q*. *acutissima* presented intermediate ecological stoichiometry (Table 3).

**TABLE 3:** Ecological stoichiometry (mean ± SD) and coefficient of variation in dominant plant species in four karst forests in Northern Guizhou Province, Southwestern China

Species	Organ	TC (mg/g)	TN (mg/g)	TP (mg/g)	C/N	C/P	N/P
*Platycarya strobilacea*	Leaf	450.38 ± 8.79	17.58 ± 1.73	0.81 ± 0.06	25.82 ± 2.48	555.21 ± 38.08	21.60 ± 1.65
*Pinus massoniana*	Leaf	530.36 ± 9.38	12.19 ± 1.37	0.83 ± 0.07	44.05 ± 5.93	646.85 ± 64.39	14.75 ± 0.93
*Albizia kalkora*	Leaf	467.83 ± 7.72	25.42 ± 2.26	0.89 ± 0.04	18.55 ± 1.98	528.69 ± 28.12	28.67 ± 2.24
*Platycladus orientalis*	Leaf	477.34 ± 15.10	10.88 ± 2.36	0.85 ± 0.15	45.40 ± 9.05	576.15 ± 80.67	12.87 ± 1.52
*Quercus fabri*	Leaf	485.88 ± 7.60	18.38 ± 1.09	0.84 ± 0.11	26.52 ± 1.67	588.89 ± 88.52	22.13 ± 2.09
*Quercus. acutissima*	Leaf	488.83 ± 15.29	18.58 ± 2.48	0.86 ± 0.12	26.74 ± 4.01	575.74 ± 86.56	21.74 ± 3.21
*Camellia japonica*	Leaf	470.00 ± 4.33	10.35 ± 1.20	0.51 ± 0.06	45.92 ± 5.58	935.76 ± 143.09	20.45 ± 2.46
*Quercus variabilis*	Leaf	497.23 ± 1.50	17.72 ± 1.23	0.85 ± 0.06	28.17 ± 2.01	584.99 ± 41.56	20.80 ± 1.17
*Lindera glauca*	Leaf	490.83 ± 9.37	20.64 ± 1.62	1.21 ± 0.14	23.88 ± 1.56	408.96 ± 41.38	17.13 ± 1.25
Mean	Leaf	484.30 ± 22.44 (4.63%)	16.86 ± 4.92 (29.21%)	0.85 ± 0.18 (20.82%)	31.67 ± 10.46 (33.02%)	600.14 ± 141.43 (23.57%)	20.02 ± 4.65 (23.22%)
*Platycarya strobilacea*	Root	445.71 ± 12.33	3.64 ± 0.68	0.14 ± 0.04	125.69 ± 22.82	3460.50 ± 1113.25	27.19 ± 4.51
*Pinus massoniana*	Root	558.82 ± 3.11	3.77 ± 0.57	0.30 ± 0.04	151.29 ± 25.63	1877.13 ± 290.49	12.47 ± 1.11
*Albizia kalkora*	Root	473.33 ± 9.15	8.82 ± 0.79	0.15 ± 0.02	54.06 ± 5.14	3232.97 ± 473.99	59.60 ± 3.91
*Platycladus orientalis*	Root	524.75 ± 5.82	2.86 ± 0.68	0.12 ± 0.02	192.82 ± 48.57	4619.41 ± 1036.50	24.25 ± 3.03
*Quercus fabri*	Root	486.78 ± 8.65	4.87 ± 0.55	0.29 ± 0.05	101.06 ± 11.19	1702.07 ± 271.95	16.83 ± 1.79
*Quercus. acutissima*	Root	479.47 ± 9.50	4.37 ± 0.18	0.29 ± 0.11	109.75 ± 3.92	1767.69 ± 452.88	16.14 ± 4.17
*Camellia japonica*	Root	509.61 ± 8.56	5.18 ± 0.70	0.48 ± 0.13	99.70 ± 12.10	1114.88 ± 292.76	11.28 ± 3.12
*Quercus variabilis*	Root	479.86 ± 5.59	5.25 ± 0.20	0.45 ± 0.09	91.55 ± 4.22	1101.01 ± 231.51	12.02 ± 2.49
*Lindera glauca*	Root	495.66 ± 8.44	7.46 ± 1.33	0.86 ± 0.27	68.23 ± 12.77	625.28 ± 212.21	9.04 ± 1.88
Mean	Root	494.89 ± 32.80 (6.63%)	5.13 ± 1.90 (37.01%)	0.34 ± 0.23 (68.14%)	110.46 ± 42.14 (38.15%)	2166.77 ± 1318.31 (60.84%)	20.98 ± 15.69 (74.81%)
*Platycarya strobilacea*	Branch	468.97 ± 5.90	4.97 ± 0.20	0.29 ± 0.07	94.42 ± 4.31	1661.25 ± 381.34	17.71 ± 4.58
*Pinus massoniana*	Branch	533.14 ± 9.42	6.28 ± 1.74	0.26 ± 0.05	91.68 ± 31.15	2129.33 ± 415.41	24.70 ± 7.61
*Albizia kalkora*	Branch	492.10 ± 8.66	13.16 ± 1.23	0.56 ± 0.10	37.66 ± 3.55	897.81 ± 167.16	23.90 ± 4.33
*Platycladus orientalis*	Branch	522.10 ± 28.93	2.80 ± 0.31	0.27 ± 0.18	188.97 ± 26.78	2430.24 ± 938.44	12.59 ± 4.36
*Quercus fabri*	Branch	461.18 ± 5.55	6.23 ± 0.92	0.23 ± 0.07	75.51 ± 12.39	2123.23 ± 512.24	28.53 ± 8.03
*Quercus. acutissima*	Branch	466.94 ± 16.30	5.74 ± 1.89	0.41 ± 0.31	89.21 ± 30.63	1501.06 ± 661.16	18.10 ± 9.35
*Camellia japonica*	Branch	492.91 ± 10.24	6.18 ± 2.06	0.53 ± 0.14	87.57 ± 30.17	1006.63 ± 334.08	11.95 ± 3.16
*Quercus variabilis*	Branch	484.35 ± 4.88	7.35 ± 1.37	0.22 ± 0.07	67.78 ± 12.69	2307.01 ± 551.35	35.78 ± 13.05
*Lindera glauca*	Branch	533.90 ± 7.78	5.83 ± 1.30	0.30 ± 0.07	96.47 ± 27.46	1930.68 ± 610.84	20.72 ± 7.01
Mean	Branch	495.06 ± 28.37 (5.73%)	6.50 ± 2.79 (42.93%)	0.34 ± 0.13 (37.44%)	92.14 ± 40.73 (44.20%)	1776.36 ± 551.28 (31.03%)	21.55 ± 7.64 (35.44%)

The TC contents of leaf, root, and branch displayed small interspecific variations, indicated by small coefficients of interspecific variation, ranging from 4.63% to 6.63% (Table 3). Whereas, other ecological stoichiometry exhibited large interspecific variations (Table 3). The ecological stoichiometry of root (37.01%–74.81%) and leaf (20.82%–33.02%) presented the largest and smallest interspecific variations, respectively, and those of branch (31.03%–44.20%) presented intermediate interspecific variations (Table 3). The maximum coefficient of the interspecific variation was RN/P (74.81%; Table 3). RP (68.14%) and RC/P (60.84%) also presented relatively large coefficients of interspecific variation (Table 3). LP presented the minimum coefficient of interspecific variation (20.82%, Table 3).

### 3.3 CWM of plant functional traits

**FIGURE 3:**
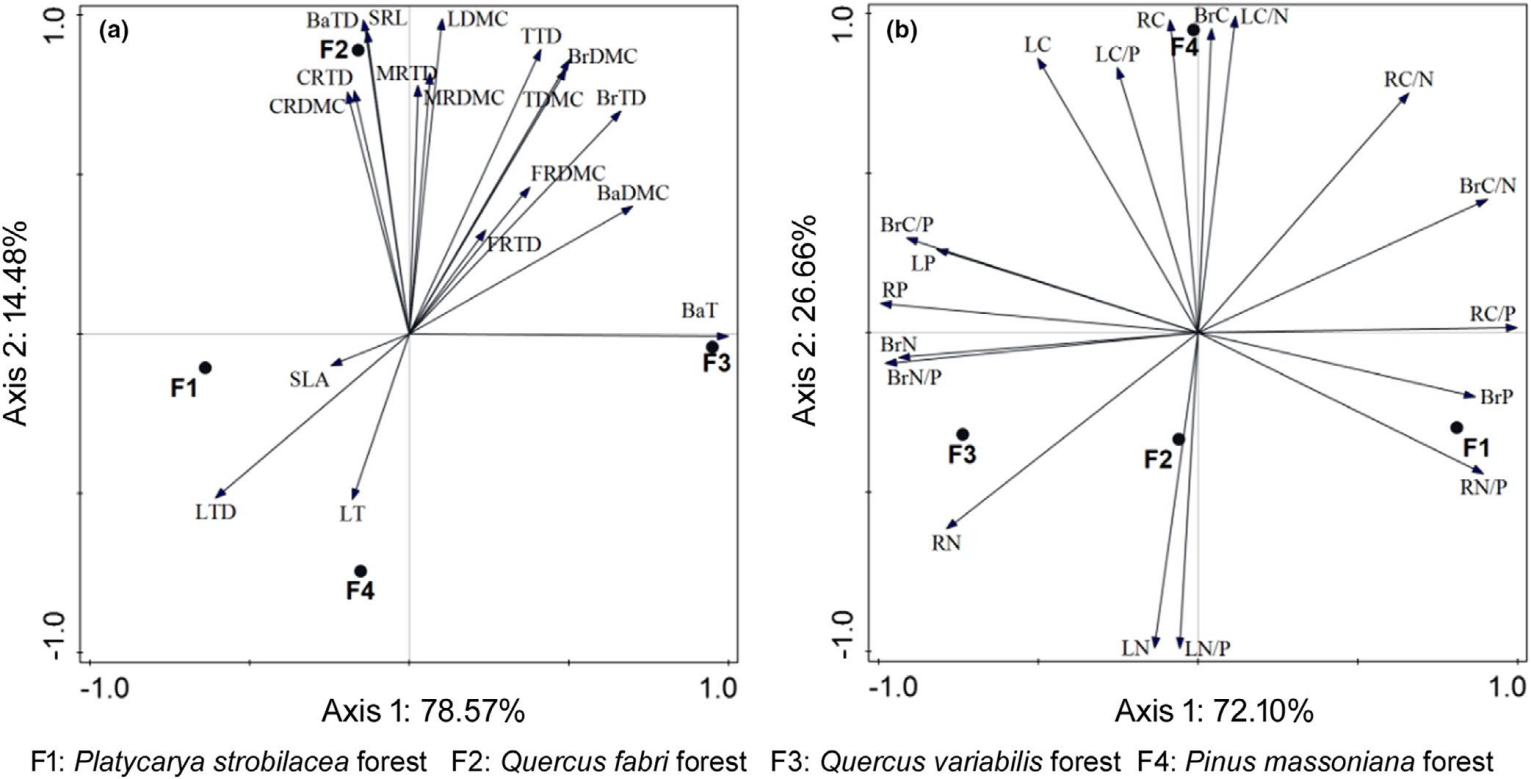
PCA showing the distribution of the morphological traits (a) and ecological stoichiometry (b) among different types of karst forest in Northern Guizhou Province, Southwestern China. Axis1 accounted for 78.57% (a) or 72.10% (b) of the variables, and Axis2 accounted for 14.48% (a) or 26.66% (b) of the variables. LT, leaf thickness; LTD, leaf tissue density; LDMC, leaf dry‐matter content; SLA, specific leaf area; CRTD, coarse root tissue density; CRDMC, coarse root dry‐matter content; MRTD, medium root tissue density; MRDMC, medium root dry‐matter content; FRTD, fine root tissue density; FRDMC, fine root dry‐matter content; SRL, fine root specific length; BrTD, branch tissue density; BrDMC, branch dry‐matter content; TTD, twig tissue density; TDMC, twig dry‐matter content; BaT, bark thickness; BaTD, bark tissue density; BaDMC, bark dry‐matter content; LC, leaf total carbon content; LN, leaf total nitrogen content; LP, leaf total phosphorus content; LC/N, leaf carbon–nitrogen ratio; LC/P, leaf carbon–phosphorus ratio; LN/P, leaf nitrogen–phosphorus ratio; RC, root total carbon content; RN, root total nitrogen content; RP, root total phosphorus content; RC/N, root carbon–nitrogen ratio; RC/P, root carbon–phosphorus ratio; RN/P, root nitrogen–phosphorus ratio; BrC, branch total carbon content; BrN, branch total nitrogen content; BrP, branch total phosphorus content; BrC/N, branch carbon–nitrogen ratio; BrC/P, branch carbon–phosphorus ratio; BrN/P, branch nitrogen–phosphorus ratio

At the community level, the *P*. *massoniana* forest presented low SRL, DMC, and TD of roots, branch, twig, and bark, LN and LN/P (Figure 3, Table S3). Among the three broad‐leaved forests, the *P*. *strobilacea* forest exhibited the highest SLA and the lowest LN, LP, and LN/P. The *Q*. *fabri* and *Q*. *variabilis* forests displayed high DMC and TD of roots, branch, twig, and bark (Figure 3, Table S3).

**FIGURE 4:**
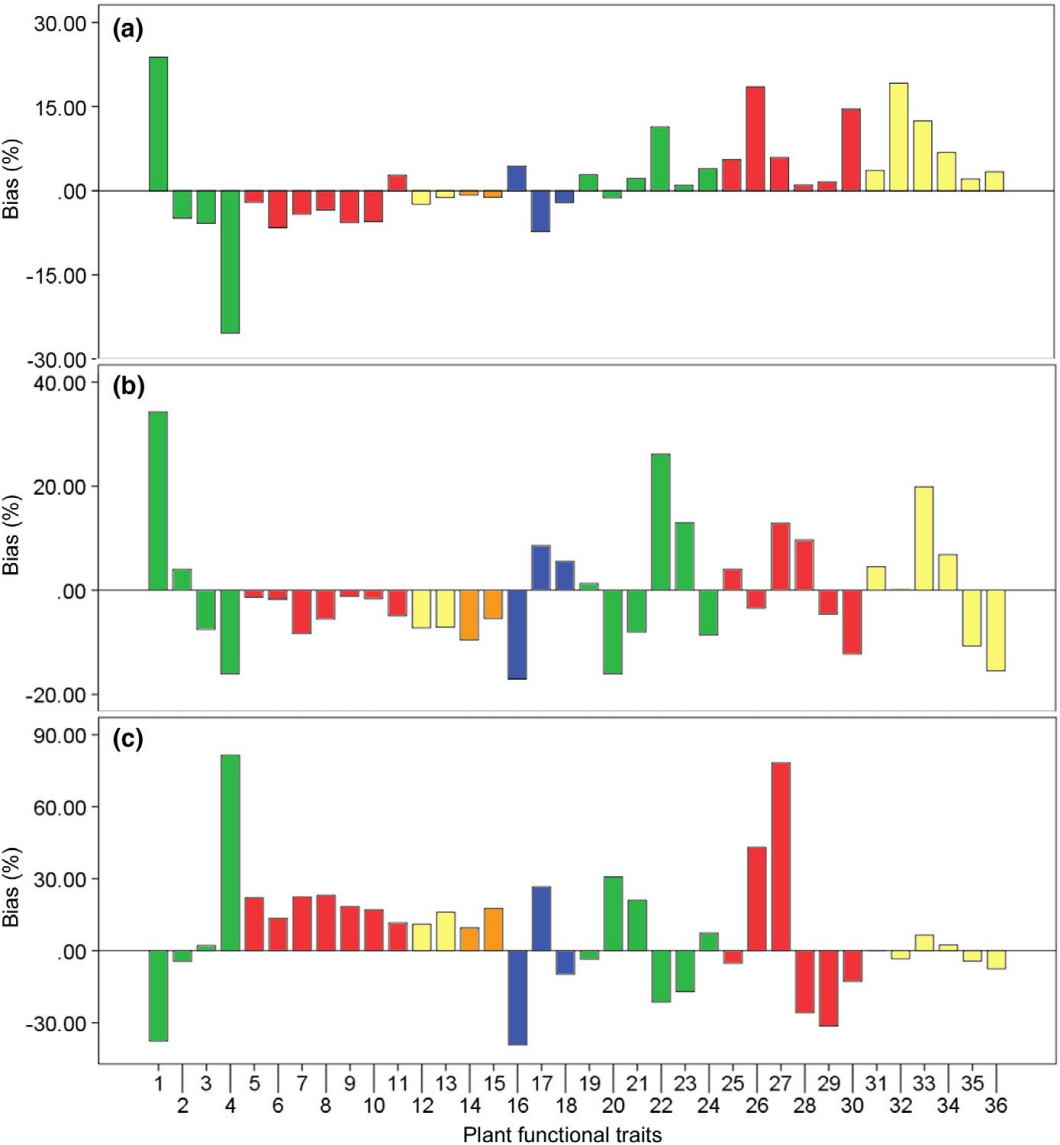
Biases of community‐weighted (CWMs) and arithmetic means of plant functional traits in three karst forests in Northern Guizhou Province, Southwestern China. Values above or below 0 mean CWMs are higher or lower than arithmetic means. Green bars: leaf traits, red bars: root traits, yellow bars: branch traits, orange bars: twig traits, blue bars: bark traits. (a) *Platycarya strobilacea* forest, (b) *Quercus fabri* forest, (c) *Pinus massoniana* forest. 1: leaf thickness, 2: leaf tissue density, 3: leaf dry‐matter content, 4: specific leaf area, 5: coarse root tissue density, 6: coarse root dry‐matter content, 7: medium root tissue density, 8: medium root dry‐matter content, 9: fine root tissue density, 10: fine root dry‐matter content, 11: fine root specific length, 12: branch tissue density, 13: branch dry‐matter content, 14: twig tissue density, 15: twig dry‐matter content, 16: bark thickness, 17: bark tissue density, 18: bark dry‐matter content, 19: leaf total carbon content, 20: leaf total nitrogen content, 21: leaf total phosphorus content, 22: leaf carbon–nitrogen ratio, 23: leaf carbon–phosphorus ratio, 24: leaf nitrogen–phosphorus ratio, 25: root total carbon content, 26: root total nitrogen content, 27: root total phosphorus content, 28: root carbon–nitrogen ratio, 29: root carbon–phosphorus ratio, 30: root nitrogen–phosphorus ratio, 31: branch total carbon content, 32: branch total nitrogen content, 33: branch total phosphorus content; 34: branch carbon–nitrogen ratio; 35: branch carbon–phosphorus ratio; 36: branch nitrogen–phosphorus ratio

When the codominant species in a forest community displayed small interspecific variations in PFTs, the CWM and the arithmetic mean of PFT values would be inevitably similar. For example, *P*. *massoniana* and *L*. *glauca* in the *P*. *massoniana* forest displayed small interspecific variations in LTD, LDMC, LC, BrC, BrN, BrC/N, and BrC/P, thus the CWM and the arithmetic mean of these PFT values were inevitably similar (Figure 4, Table S3). Besides, when the product of higher (compared with the CWM) trait values and its/their relative biomass of species and the product of lower (compared to the CWM) trait values and its/their relative biomass of species counterbalanced, the CWM and the arithmetic mean of PFT values might be coincidently similar. For example, in the *P*. *strobilacea* forest, BaT, LN, and LN/P presented large interspecific variations, while the CWM and the arithmetic mean of these PFT values were similar resulting from the counterbalances of higher and lower trait values (Figure 4, Table S3). Otherwise, the CWM of PFT values were preferential to represent the trait characteristics at the community level (Figure 4, Table S3).

## 4 DISCUSSION

Few PFT studies have been conducted in karst geomorphology compared with normal geomorphologies in Southern China, and most of the existing studies only focus on leaf traits, such as LA, LT, LDMC, SLA, and LN/P (Jiang et al., 2016; Liu et al., 2014, 2015, 2019; Pang et al., 2019; Xi et al., 2011). The characteristics of root, stem, branch, and twig traits are rarely investigated (Liu et al., 2019; Pi et al., 2017; Yang et al., 2020; Zhong et al., 2018), and those of bark traits are never reported. The present study comprehensively investigates the characteristics of 18 morphological traits of leaf, root, branch, twig, and bark and the C:N:P ecological stoichiometry of leaf, root, and branch of dominant species in four typical forests growing in a trough‐valley karst watershed in Southwestern China. Such a study can fill the blanks in the PFT studies in karst regions in Southern China.

The interspecific variations in PFTs are the main research contents of PFT studies because interspecific variations play a dominant role in the variations in PFTs. In the present study, the coefficients of interspecific variation in the morphological traits range from 9.52% to 96.89% (average coefficient of interspecific variation of the 18 morphological traits = 26.88%), and those of C:N:P ecological stoichiometry range from 4.63% to 74.81% (average coefficient of interspecific variation of the 18 ecological stoichiometry = 34.27%) (Tables 2 and 3). Average interspecific trait variations align with values found in previous studies (Jiang et al., 2016; Liu et al., 2014, 2015; Xi et al., 2011). Leaf traits present large and branch and twig traits present small interspecific variations in previous studies and the present study. Interspecific variations in root traits often exhibit high uncertainties due to complex and diverse belowground habitats (Comas & Eissenstat, 2004; Westoby & Wright, 2006). In the present study, the morphological traits of roots display small interspecific variations compared to those of leaf, whereas the root C:N:P ecological stoichiometry displays large interspecific variations compared to leaf and branch C:N:P ecological stoichiometry. Minimal attention has been paid to bark traits. We have investigated the BaT, BaTD, and BaDMC of species in karst vegetation and found that bark traits present large interspecific variations compared to roots, branch, and twig traits.

Overall, most PFT studies worldwide focus on several dominant or model species and ignore the complex species composition and the community structure in natural plant communities. Thus, whether the conclusions derived from such studies are applicable to complex natural plant communities remains to be verified (Díaz et al., 2016; Wright et al., 2004). The connection of individual‐level PFTs with community structures, processes, and functions becomes a hot and difficult topic in this research field (Kunstler et al., 2016; Reichstein et al., 2014). In recent years, some plant ecologists successfully extrapolated PFT characteristics from the organ and the species levels to community and ecosystem levels on the basis of relative biomass or individual number (especially the former) of species in a plant community (Ali et al., 2017; Zhang et al., 2018). Karst forests are known for their rich species composition and high interspecific variations in PFTs (compared to non‐karst forests in the same climate zone). Thus, the direct use of the arithmetic mean traits to represent the community traits is inappropriate. In the present study, we have calculated the species biomass‐weighted mean community traits and found that CWM traits are preferential to represent the traits at the community level, which are indicated by high biases between CWM and arithmetic mean traits (Figure 4, Table S3).

PFTs are jointly determined by genetic factors and environmental conditions (Weiher & Keddy, 1995). In the present study, all selected plants and forests are located in the same karst watershed and share similar habitats and resource conditions. The *P*. *massoniana* forest (the artificial forest) presents the lowest community values of DMC and TD of roots, branch, twig, and bark. The special trait assembly indicates that *P*. *massoniana* is a fast‐growing species. However, both needle‐leaved and broad‐leaved species and forests in the karst geomorphology present low SLA and high DMC and TD of roots, branch and twig at the species and the community levels compared with those in normal geomorphologies in the same climate zone (Chen et al., 2016; Guo et al., 2019; Tang et al., 2016; Wang et al., 2015; Zhong et al., 2018). The trait assembly of low SLA and high DMC and TD of roots, branch, and twig at the community level in karst forests are beneficial to reduce transpiration and water loss and increase the nutrient storage for adaptation to harsh karst habitats with high temperature, water shortage, and shallow soils (Pang et al., 2019; Yang et al., 2020; Zhong et al., 2018).

The four karst forests have slightly lower community LN contents (12.54–17.72 mg g^−1^) and significantly lower LP contents (0.82–0.85 mg g^−1^) than plants in China (LN: 18.6 mg g^−1^; LP: 1.21 mg g^−1^) and in the world (20.09 and 1.77 mg g^−1^), indicating that karst forests and plants present low LN and LP contents (especially the latter) (Han et al., 2005; Reich & Oleksyn, 2004). According to Koerselman and Meuleman (1996) and Tessier and Raynal (2003), LN/P < 14 indicates N limitation, LN/P > 16 indicates P limitation, and 14 < LN/P <16 indicates a colimitation of N and P. The LN/P value (14.85) of *P*. *massoniana* forest suggests N and P colimitation, and the LN/P values (20.26–21.64) of the three other broad‐leaved forests (20.26–21.64) point to P limitation.

The forest restoration of degraded vegetation, such as grasslands, tussocks, and shrublands created by intensive human disturbances, has become a formidable task in karst regions in Southwestern China, and the increases in the biodiversity and the C storage are often used to evaluate the restoration success (Liu et al., 2011; Ni et al., 2015). PFTs and the trait‐based community ecology theory provide another pathway to predict the success of restoration efforts and the prospects of local vegetation restoration (Hedberg et al., 2013; Lavorel & Garnier, 2002; Pywell et al., 2003; Roberts et al., 2010; Sandel et al., 2011). In the present study, the *P*. *massoniana* forest is a fast‐growing forest, which can rapidly increase the local vegetation coverage and the C storage. The *P*. *strobilacea* forest presents relatively high SLA and low DMC and TD of roots, branch, and twig (compared with *Q*. *fabri* forest and *Q*. *variabilis* forest). It allocates increased resources to growth and is in the early succession stage. The trait assembly of the *Q*. *fabri* and the *Q*. *variabilis* forests indicates the allocation of increased resources to survive and the best adaptation to harsh karst habitats in this watershed. Understanding the adaptation strategy of karst forests would help to restore forests in karst regions in Southwestern China.

## 5 CONCLUSIONS

Overall, the CWMs rather than the arithmetic means of PFTs were preferential to represent the trait characteristics at the community level. From the view of plant functional traits at the community level, karst forests adopt a conservative survival strategy. Considering plant trait assembly and resource utilization would promote ecological restoration in karst regions in Southwestern China.

## CONFLICT OF INTEREST

The authors declare no conflict of interest.

## AUTHOR CONTRIBUTIONS

**Libin Liu:** Conceptualization (equal); Data curation (equal); Formal analysis (equal); Funding acquisition (equal); Investigation (equal); Methodology (equal); Writing – original draft (equal); Writing – review & editing (equal). **Jing Hu:** Data curation (equal); Formal analysis (equal). **Xinyao Chen:** Data curation (equal); Formal analysis (equal). **Xin Xu:** Data curation (equal); Formal analysis (equal); Investigation (lead). **Yong Yang:** Data curation (equal); Formal analysis (equal); Investigation (lead). **Jian Ni:** Conceptualization (lead); Funding acquisition (equal); Writing – original draft (equal); Writing – review & editing (equal).

## Supporting information

Table S1‐S3

## Data Availability

The data are available in the manuscript and supplementary materials.
