# Supplementary material for: Adaptation strategy of karst forests: Evidence from the community‐weighted mean of plant functional traits
Source: Ecol Evol. 2022 Mar 1;12(3):e8680. doi: 10.1002/ece3.8680 (PMC8888248; doi:10.1002/ece3.8680)
Supplement: Supplementary file 1 — Table S1‐S3 [file ECE3-12-e8680-s001.doc]

**Supplementary Materials: Adaptation strategy of karst forests: Evidence from the community-weighted mean of plant functional traits**

Libin Liu | Jing Hu | Xinyao Chen | Xin Xu | Yong Yang | Jian Ni

**TABLE S1** Biomass allometric models of karst forests in Northern Guizhou Province, Southwestern China. The units of biomass, *D* and height are g, cm and cm, respectively, in the study of Liu et al. (2009); g, cm and m, respectively, in the studies of Chen et al. (1993) and Liu et al. (2018) and kg, cm and m, respectively, in other studies.

| Species | Biomass allometric models | Correlation coefficient | References |
| --- | --- | --- | --- |
| *Platycarya strobilacea* | Wa=1.9611×(D^2^H)^0.8921^  Wr=0.0007×(D^2^H)^2^+0.611×(D^2^H)+5228.5 | 0.9886  0.4689 | Liu et al., 2009; 2018 |
| *Pinus massoniana* | Wt=0.428×D^2.009^ | 0.991 | Du et al., 2014 |
| *Albizia kalkora* | logWs=0.75995×log(D^2^H)-0.75237  logWbr=0.69997×log(D^2^H)-0.93934  logWl=0.53231×log(D^2^H)-0.96854  Wr=13.602×D^2^+75.709×D+1393.9 | 0.948  0.959  0.915  0.4968 | Deng et al., 2000; Liu et al., 2018 |
| *Platycladus orientalis* | Ws=0.1809×(D^2^H)^0.6682^  Wbr=0.1009×(D^2^H)^0.6497^  Wl=0.1163×(D^2^H)^0.5385^  Wr=0.036+0.0098×(D^2^H) | 0.99  0.99  0.98  / | Wang et al., 2018 |
| *Quercus fabri* | Ws=0.020×(D^2^H)^0.990^  Wba=0.020×(D^2^H)^0.770^  Wbr=0.005×(D^2^H)^0.997^  Wl=0.007×(D^2^H)^0.777^  Wr=0.020×(D^2^H)^0.821^ | 0.969  0.896  0.870  0.747  0.879 | Shen et al., 2011 |
| *Quercus acutissima* | Ws=0.56310×(D^2^H)^0.6180^  Wbr=0.00681×(D^2^H)^0.9481^  Wl=0.01823×(D^2^H)^0.6241^  Wr=0.26852×(D^2^H)^0.5477^ | 0.91575  0.87531  0.81267  0.84960 | Yang, 2004 |
| *Quercus variabilis* | logWs=-0.544023+0.679572×log(D^2^H)  logWba=-0.824562+0.589619×log(D^2^H)  logWbr=-2.560986+1.109206×log(D^2^H)  logWl=-2.003840+0.746017×log(D^2^H)  logWr=-0.264502+0.517306×1og(D^2^H) | 0.9969  0.9983  0.9750  0.9851  0.9986 | Liu et al., 1998 |
| *Lindera glauca* | Wt=86.589× (D^2^H)^0.8699^ | / | Chen et al., 1993 |
| *Cinnamomum camphora*, *Cinnamomum bodinieri* | Wt=1.728+0.015×(D^2^H) | 0.938 | He et al., 2007 |
| *Cunninghamia lanceolata* | Wt=0.096×D^2.41^ | 0.993 | Du et al., 2014 |
| *Tilia kueichouensis* | Wt=0.023×(D^2^H)^1.01^ | 0.985 | Dai et al., 2013 |
| Other species | Wa=0.5834×(D^2^H)-8.151 (1.0≤D≤5.0 cm)  Wa=2.0141×(D^2^H)0.889 (D>5.0 cm)  Wr=13.602×D^2^+75.709×D+1393.9 (trees)  Wr=21.84×D^2^+50.575×D-1.8387 (shrubs) | 0.9228  0.9584  0.4968  0.6993 | Liu et al., 2009; 2018 |

Wa, aboveground biomass; Wr, root biomass; Wt, total biomass; Ws, stem biomass; Wbr, branch biomass; Wl, leaf biomass; Wba, bark biomass

**References**

Chen, Q. C. (1993). Primary productivity of evergreen broad-leaved forest ecosystem. Hangzhou University Press, Hangzhou, China.

Dai, H. J., He, H. J., Zhao, X. H., Zhang, C. Y., Wang, J. S., & Yang, S. (2013). Biomass allocation patterns and allometric models of two dominant tree species in broad-leaved and Korean pine mixed forest. *Chinese Journal of Applied & Environmental Biology*, 19, 718–722.

Deng, S. J., Liao, L. P., Wang, S. L., Gao, H., & Lin, B. (2000). Bioproductivity of *Castanopsisi hysrix* – *Cyclobalanopsis glauca* – *Machilus pauhoi* community in Huitong, Hunan. *Chinese Journal of Applied Ecology*, 11, 651–654.

Du, H., Zeng, F. P., Wang, K. L., Song, T. Q., Wen, Y. G., Li, C. G., Peng, W. X., Liang, H. W., Zhu, H. G., & Zeng, Z. X. (2014). Dynamics of biomass and productivity of three major plantation types in southern China. *Acta Ecologica Sinica*, 34, 2712–2724.

He, H. Z., Huang, L. H., Duan, X., & He, R. K. (2007). Study on biomass in main afforestation tree species of the second ring forest-belt of Guiyang. *Guizhou Science*, 25, 33–39.

Liu, C. C., Wei, Y. F., Liu, Y. G., & Guo, K. (2009). Biomass of canopy and shrub layers of karst forest in Puding, Guizhou, China. *Chinese Journal of Plant Ecology*, 33, 698–705.

Liu, L. B., Zhong, Q. L. & Ni, J. (2018). Allometric function-based root biomass estimate of woody plants in a karst evergreen and deciduous broadleaf and mixed forest in central Guizhou Province, southwestern China. *Acta Ecologica Sinica*, 38, 8726–8732.

Liu, Y. C., Wu, M. Z., Guo, Z. M., Jiang, Y. X., & Liu, S. R. (1998). Biomass and net productivity of *Quercus variabilis* forest in Baotianman Natural Reserve. *Chinese Journal of Applied Ecology*, 9, 569–574.

Shen, Y., Tian, D. L., Yan, W. D., & Xiao, Y. (2011). Biomass and its distribution of natural secondary *Quercus fabri* + *Sassafras tsumu* + *Cunninghamia lanceolata* community in Yuanling County, Hunan Province. *Journal of Central South University of Forestry & Technology*, 31, 44–51.

Wang, Y. F., Liu, L., Li, Z. C., Shi, X. F., Yang, X. Y., & Shangguan, Z. P. (2018). Storage of carbon and nitrogen in Q*uercus* and *Platycladus orientalis* plantations at different ages in the hilly area of western Henan Province, China. *Chinese Journal of Applied Ecology*, 29, 25–32.

Yang, T. (2004). The investigation and study to the natural secondary mixed forest’s biomass and the root system’s spreading character of the oak and the horse-tail pine. *Journal of Xinyang Agricultural College*, 14, 4–6,9.

**TABLE S2** Plant functional traits and their abbreviations, units and calculations.

| Plant functional trait | Abbreviation | Unit | Calculation |
| --- | --- | --- | --- |
| Leaf tissue density | LTD | g/cm^3^ | Leaf dry mass/volume |
| Leaf dry matter content | LDMC | g/g | Leaf dry mass/fresh mass |
| Specific leaf area | SLA | cm^2^/g | Leaf area/leaf dry mass |
| Coarse root tissue density | CRTD | g/cm^3^ | Coarse root dry mass/volume |
| Coarse root dry matter content | CRDMC | g/g | Coarse root dry mass/fresh mass |
| Medium root tissue density | MRTD | g/cm^3^ | Medium root dry mass/volume |
| Medium root dry matter content | MRDMC | g/g | Medium root dry mass/fresh mass |
| Fine root tissue density | FRTD | g/cm^3^ | Fine root dry mass/volume |
| Fine root dry matter content | FRDMC | g/g | Fine root dry mass/fresh mass |
| Fine root specific length | SRL | cm/g | Fine root length/dry mass |
| Branch tissue density | BrTD | g/cm^3^ | Branch dry mass/volume |
| Branch dry matter content | BrDMC | g/g | Branch dry mass/fresh mass |
| Twig tissue density | TTD | g/cm^3^ | Twig dry mass/volume |
| Twig dry matter content | TDMC | g/g | Twig dry mass/fresh mass |
| Bark tissue density | BaTD | g/cm^3^ | Bark dry mass/volume |
| Bark dry matter content | BaDMC | g/g | Bark dry mass/fresh mass |

**TABLE S3** Comparison of community-weighted and arithmetic means of plant functional traits in three karst forests in Northern Guizhou Province, Southwestern China. Biases ≥ 15% are highlighted in bold. LT: leaf thickness, LTD: leaf tissue density, LDMC: leaf dry-matter content, SLA: specific leaf area, CRTD: coarse root tissue density, CRDMC: coarse root dry-matter content, MRTD: medium root tissue density, MRDMC: medium root dry-matter content, FRTD: fine root tissue density, FRDMC: fine root dry-matter content, SRL: fine root specific length, BrTD: branch tissue density, BrDMC: branch dry-matter content, TTD: twig tissue density, TDMC: twig dry-matter content, BaT: bark thickness, BaTD: bark tissue density, BaDMC: bark dry-matter content, LC: leaf total carbon content, LN: leaf total nitrogen content, LP: leaf total phosphorus content, LC/N: leaf carbon–nitrogen ratio, LC/P: leaf carbon–phosphorus ratio, LN/P: leaf nitrogen–phosphorus ratio, RC: root total carbon content, RN: root total nitrogen content, RP: root total phosphorus content, RC/N: root carbon–nitrogen ratio, RC/P: root carbon–phosphorus ratio, RN/P: root nitrogen–phosphorus ratio, BrC: branch total carbon content, BrN: branch total nitrogen content, BrP: branch total phosphorus content, BrC/N: branch carbon–nitrogen ratio, BrC/P: branch carbon–phosphorus ratio, BrN/P: branch nitrogen–phosphorus ratio.

| Plant functional traits | *P. strobilacea* forest | | | *Q. fabri* forest | | | *P. massoniana* forest | | |
| --- | --- | --- | --- | --- | --- | --- | --- | --- | --- |
|  | Community-weighted mean | Arithmetic mean | Bias (%) | Community-weighted mean | Arithmetic mean | Bias (%) | Community-weighted mean | Arithmetic mean | Bias (%) |
| LT (mm) | 0.20 | 0.25 | **23.78** | 0.26 | 0.34 | **34.25** | 0.40 | 0.25 | **−37.66** |
| LTD (g/cm^3^) | 0.53 | 0.50 | −4.88 | 0.52 | 0.54 | 4.01 | 0.60 | 0.57 | −4.37 |
| LDMC (g/g) | 0.46 | 0.43 | −5.80 | 0.56 | 0.52 | −7.50 | 0.39 | 0.40 | 2.12 |
| SLA (cm^2^/g) | 120.43 | 89.81 | **−25.42** | 94.17 | 79.03 | **−16.08** | 93.34 | 169.33 | **81.41** |
| CRTD (g/cm^3^) | 0.54 | 0.53 | −2.06 | 0.53 | 0.53 | −1.34 | 0.38 | 0.47 | **21.95** |
| CRDMC (g/g) | 0.52 | 0.49 | −6.60 | 0.51 | 0.50 | −1.73 | 0.42 | 0.47 | 13.56 |
| MRTD (g/cm^3^) | 0.51 | 0.49 | −4.15 | 0.54 | 0.49 | −8.34 | 0.32 | 0.39 | **22.35** |
| MRDMC (g/g) | 0.53 | 0.51 | −3.45 | 0.53 | 0.50 | −5.50 | 0.38 | 0.47 | **23.00** |
| FRTD (g/cm^3^) | 0.64 | 0.61 | −5.62 | 0.52 | 0.51 | −1.20 | 0.40 | 0.47 | **18.41** |
| FRDMC (g/g) | 0.60 | 0.56 | −5.46 | 0.56 | 0.55 | −1.59 | 0.46 | 0.54 | **17.03** |
| SRL (cm/g) | 18.06 | 18.56 | 2.76 | 26.79 | 25.48 | −4.87 | 16.59 | 18.51 | 11.57 |
| BrTD (g/cm^3^) | 0.55 | 0.53 | −2.39 | 0.67 | 0.62 | −7.20 | 0.46 | 0.51 | 11.01 |
| BrDMC (g/g) | 0.54 | 0.53 | −1.21 | 0.63 | 0.59 | −7.06 | 0.46 | 0.53 | **15.97** |
| TTD (g/cm^3^) | 0.52 | 0.52 | −0.72 | 0.65 | 0.59 | −9.52 | 0.44 | 0.48 | 9.51 |
| TDMC (g/g) | 0.52 | 0.51 | −1.15 | 0.66 | 0.63 | −5.37 | 0.50 | 0.58 | **17.57** |
| BaT (mm) | 4.75 | 4.95 | 4.39 | 7.75 | 6.43 | **−17.06** | 7.91 | 4.81 | **−39.18** |
| BaTD (g/cm^3^) | 0.45 | 0.42 | −7.27 | 0.59 | 0.64 | 8.53 | 0.33 | 0.41 | **26.54** |
| BaDMC (g/g) | 0.52 | 0.53 | −2.13 | 0.62 | 0.66 | 5.56 | 0.58 | 0.53 | −9.79 |
| LC (mg/g) | 468.32 | 481.476 | 2.81 | 487.50 | 493.76 | 1.28 | 528.69 | 510.59 | −3.42 |
| LN (mg/g) | 16.72 | 16.51 | −1.25 | 17.72 | 14.87 | **−16.08** | 12.54 | 16.41 | **30.84** |
| LP (mg/g) | 0.82 | 0.84 | 2.21 | 0.83 | 0.76 | −8.04 | 0.84 | 1.02 | **21.02** |
| LC/N | 30.03 | 33.45 | 11.40 | 28.39 | 35.80 | **26.14** | 43.19 | 33.96 | **−21.37** |
| LC/P | 571.21 | 576.72 | 0.97 | 607.68 | 686.81 | 13.02 | 636.81 | 527.91 | **−17.10** |
| LN/P | 20.26 | 19.47 | 3.91 | 21.64 | 19.77 | −8.64 | 14.85 | 15.94 | 7.31 |
| RC (mg/g) | 474.50 | 500.65 | 5.51 | 488.98 | 508.67 | 4.03 | 556.15 | 527.24 | −5.20 |
| RN (mg/g) | 4.03 | 4.77 | **18.47** | 4.71 | 4.55 | −3.43 | 3.93 | 5.61 | **43.01** |
| RP (mg/g) | 0.17 | 0.18 | 5.94 | 0.30 | 0.34 | 12.90 | 0.33 | 0.58 | **78.28** |
| RC/N | 129.66 | 130.97 | 1.01 | 105.28 | 115.45 | 9.66 | 147.79 | 109.76 | **−25.73** |
| RC/P | 3245.68 | 3297.50 | 1.60 | 1692.59 | 1615.44 | −4.56 | 1824.27 | 1251.20 | **−31.41** |
| RN/P | 26.95 | 30.88 | 14.58 | 16.15 | 14.18 | −12.21 | 12.32 | 10.76 | −12.72 |
| BrC (mg/g) | 486.55 | 481.48 | 3.60 | 467.38 | 488.54 | 4.53 | 533.17 | 533.52 | 0.07 |
| BrN (mg/g) | 5.71 | 6.80 | **19.14** | 6.10 | 6.11 | 0.16 | 6.26 | 6.05 | −3.27 |
| BrP (mg/g) | 0.31 | 0.35 | 12.42 | 0.30 | 0.36 | **19.88** | 0.26 | 0.28 | 6.46 |
| BrC/N | 96.60 | 103.18 | 6.81 | 80.50 | 85.99 | 6.82 | 91.88 | 94.08 | 2.38 |
| BrC/P | 1742.96 | 1779.66 | 2.11 | 1892.59 | 1690.06 | −10.70 | 2120.94 | 2030.00 | −4.29 |
| BrN/P | 19.08 | 19.72 | 3.37 | 24.63 | 20.82 | **−15.48** | 24.54 | 22.71 | −7.43 |
